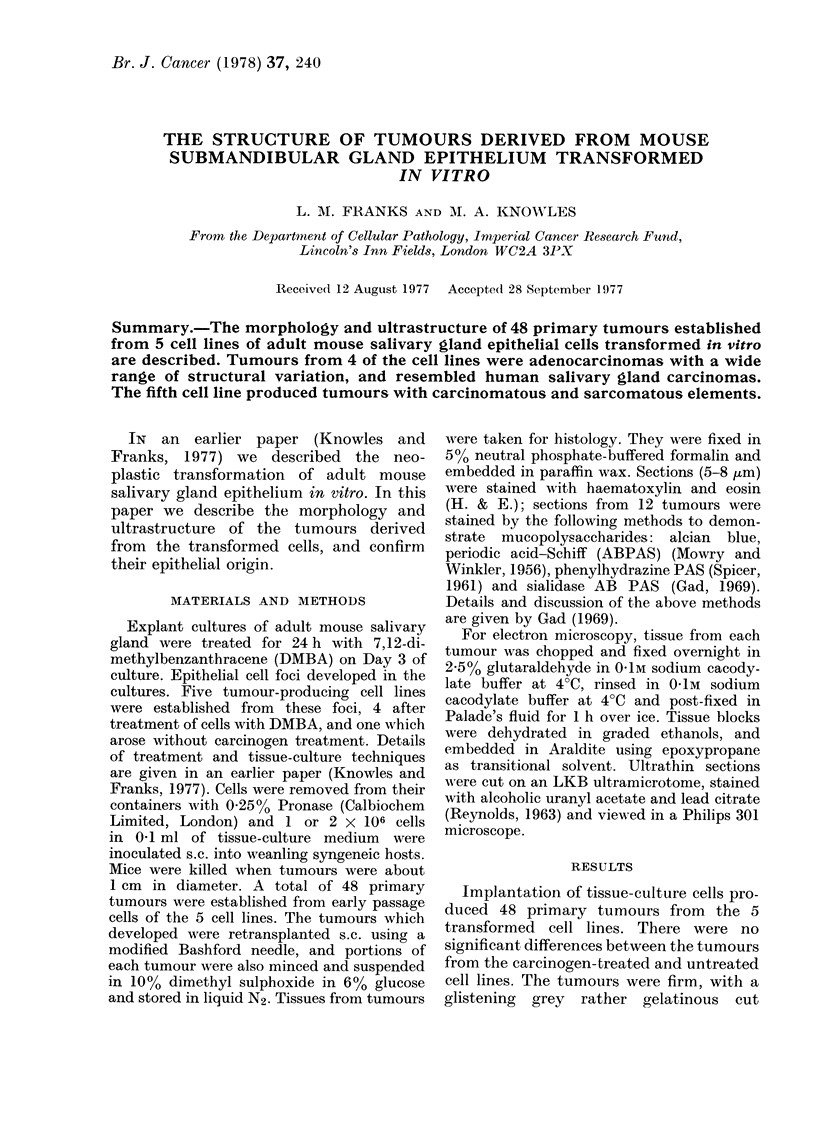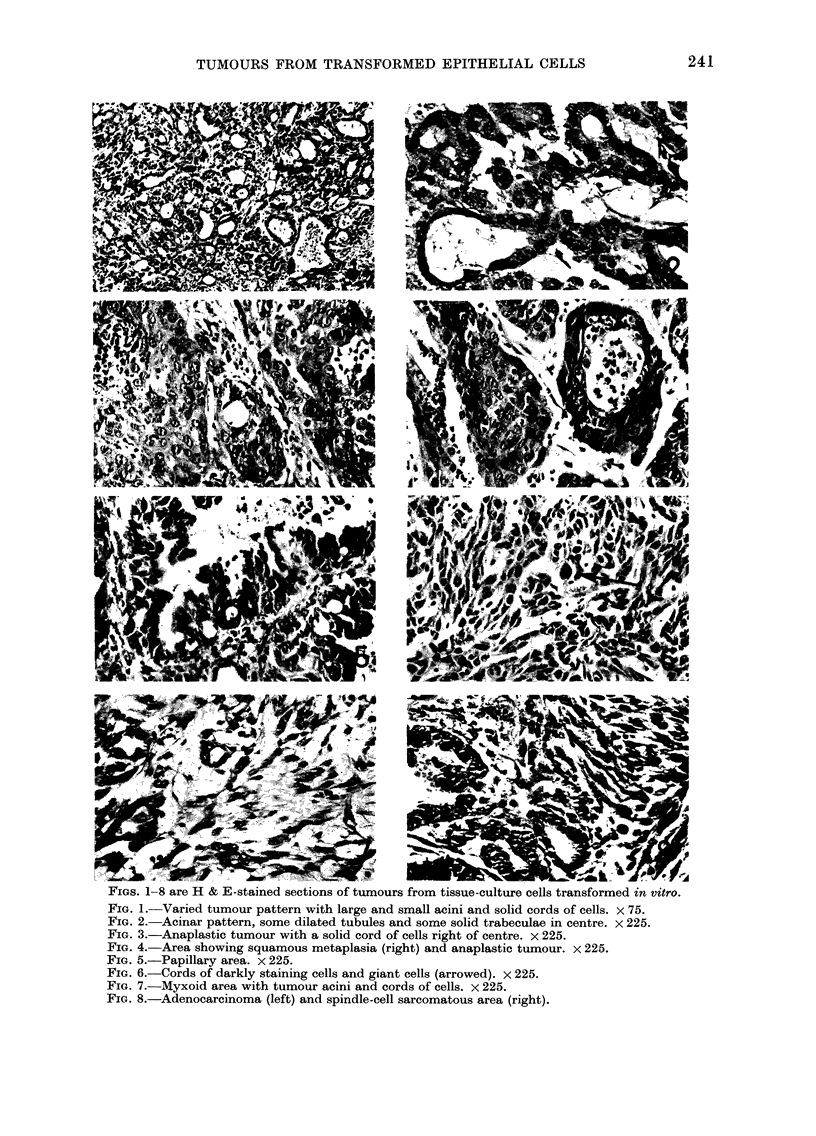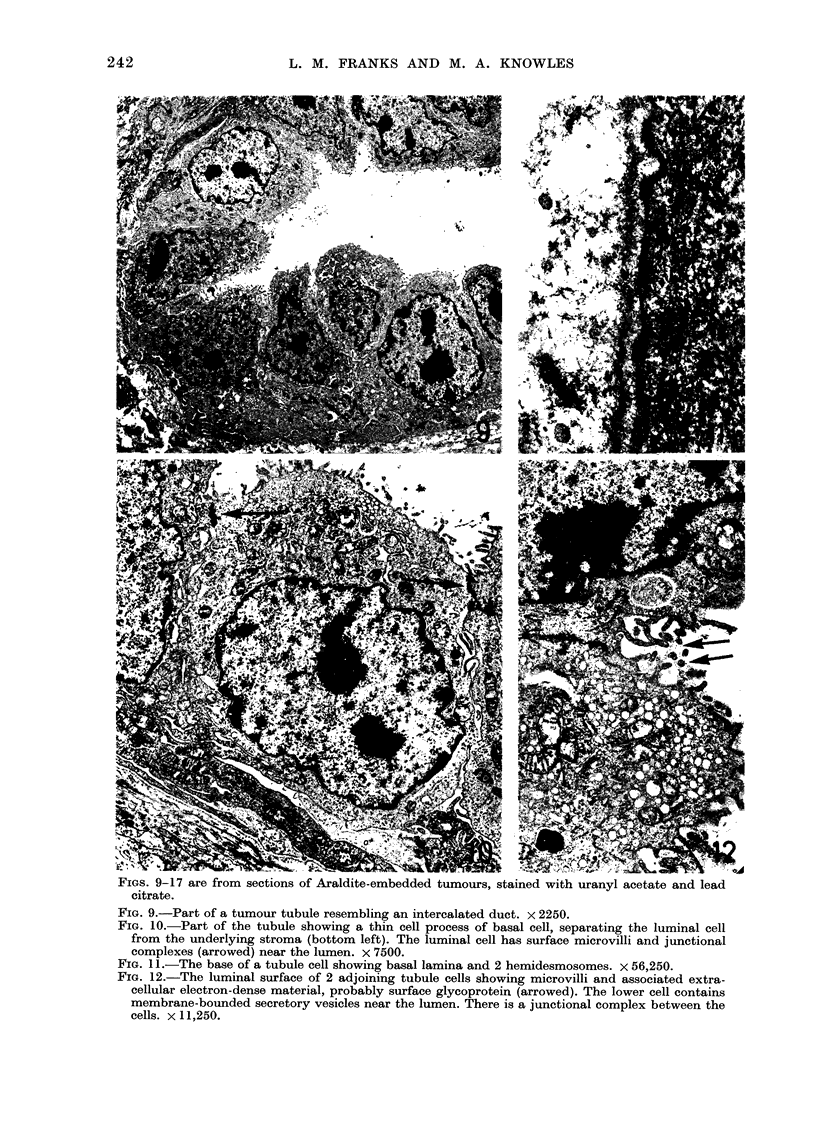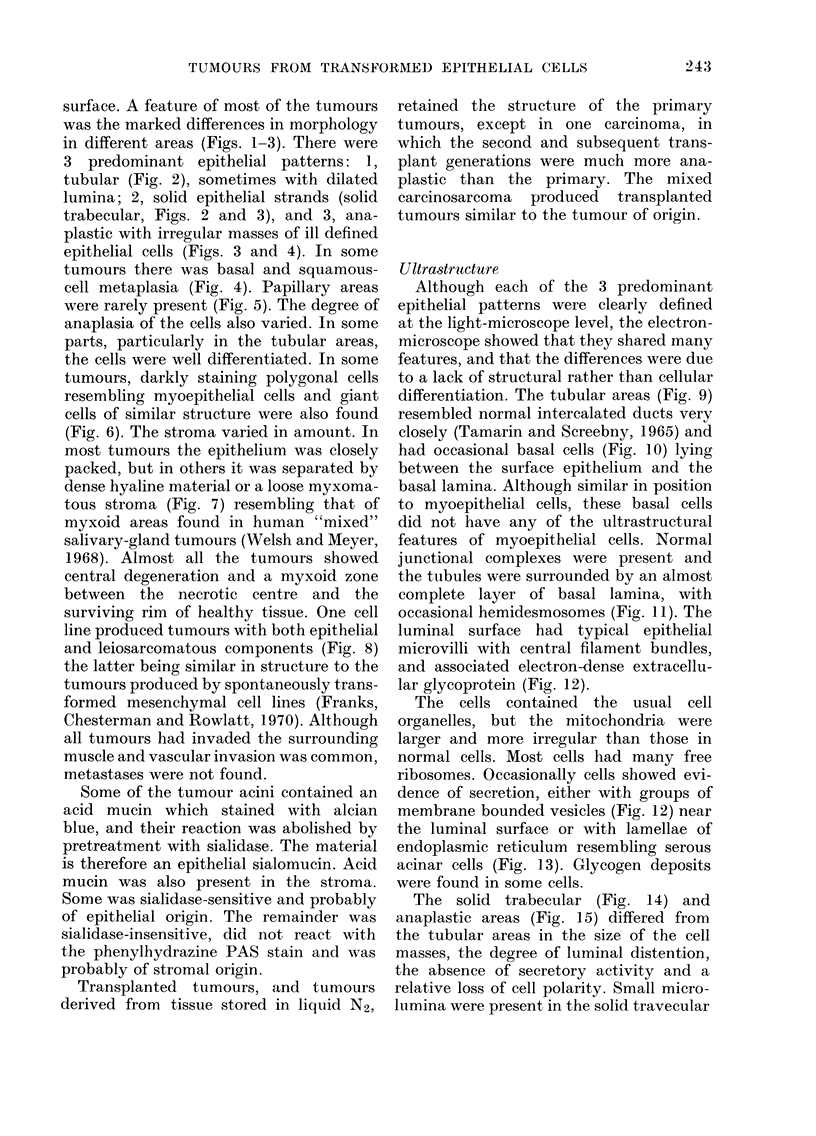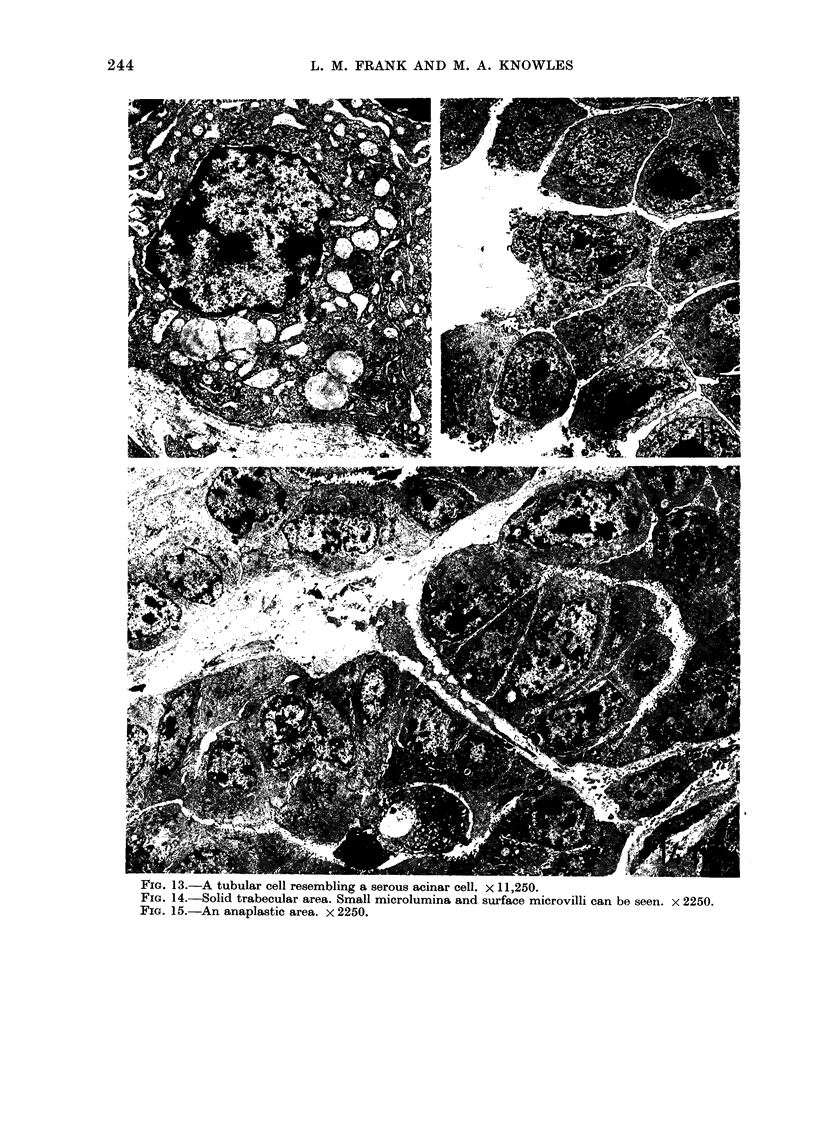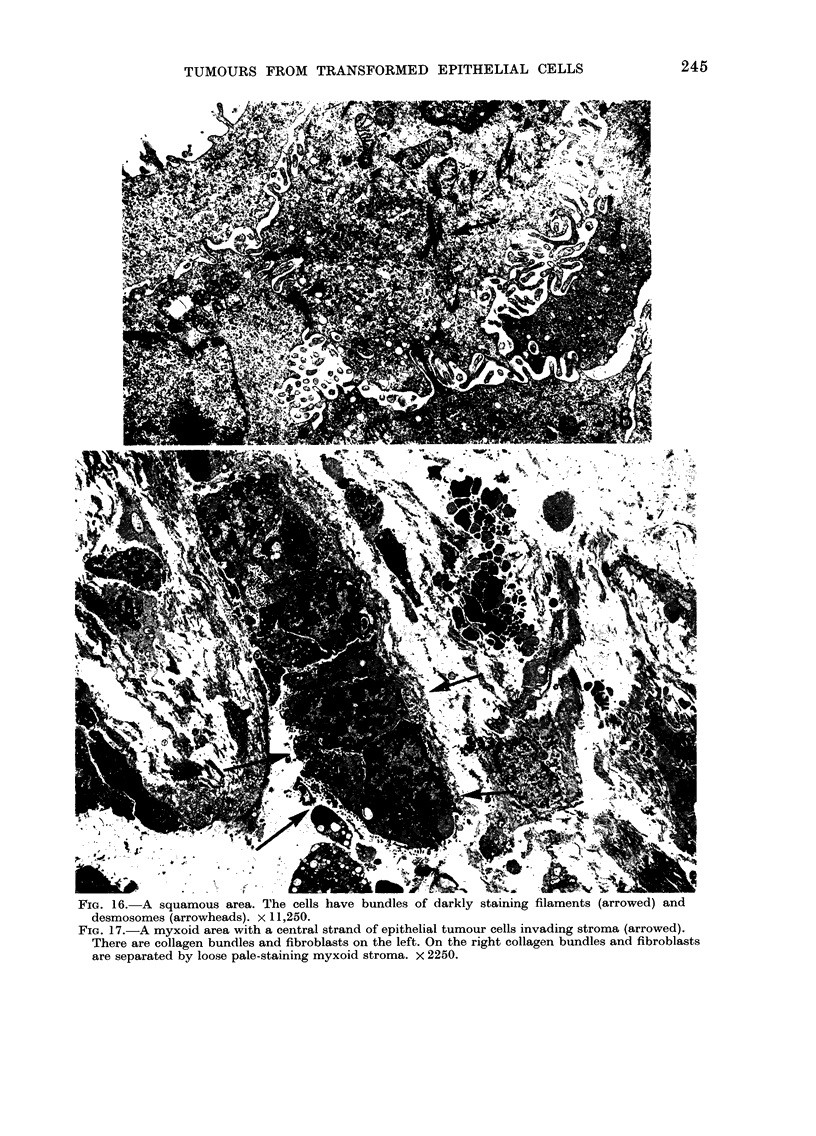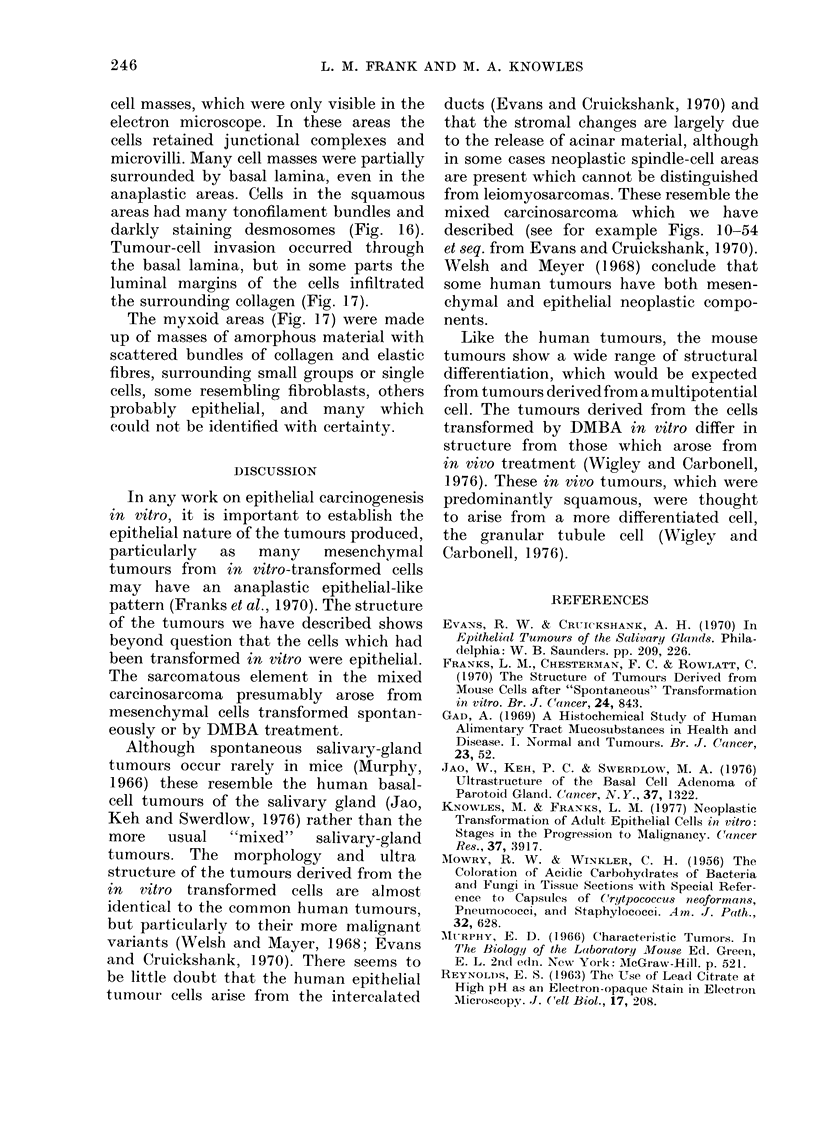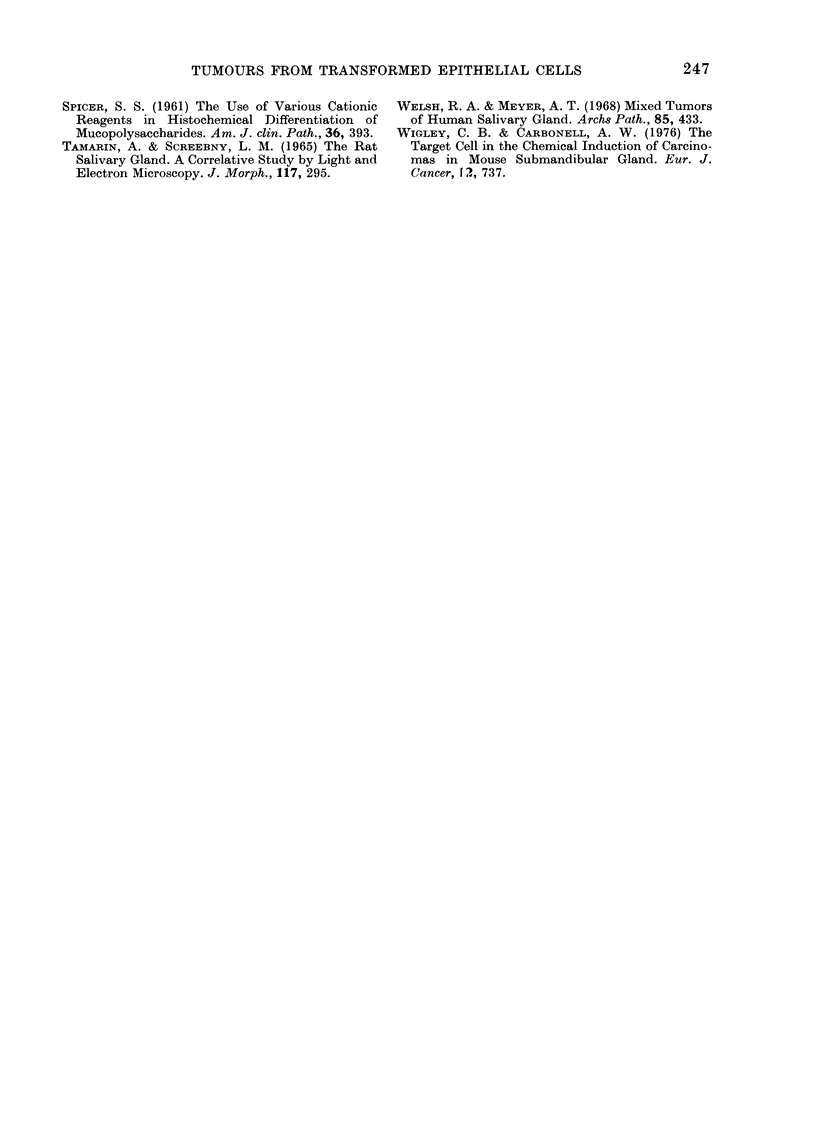# The structure of tumours derived from mouse submandibular gland epithelium transformed in vitro.

**DOI:** 10.1038/bjc.1978.32

**Published:** 1978-02

**Authors:** L. M. Franks, M. A. Knowles

## Abstract

**Images:**


*Br. J. Cancer* (1978) **37**, 240

### THE STRUCTURE OF TUMOURS DERIVED FROM MOUSE SUBMANDIBULAR GLAND EPITHELIUM TRANSFORMED *IN VITRO*

L. M. FRANKS AND M. A. KNOWLES

*From the Department of Cellular Pathology, Imperial Cancer Research Fund, Lincoln's Inn Fields, London WC2A 3PX*

Received 12 August 1977 Accepted 28 September 1977

**Summary.—The morphology and ultrastructure of 48 primary tumours established from 5 cell lines of adult mouse salivary gland epithelial cells transformed *in vitro* are described. Tumours from 4 of the cell lines were adenocarcinomas with a wide range of structural variation, and resembled human salivary gland carcinomas. The fifth cell line produced tumours with carcinomatous and sarcomatous elements.**

IN an earlier paper (Knowles and Franks, 1977) we described the neoplastic transformation of adult mouse salivary gland epithelium *in vitro*. In this paper we describe the morphology and ultrastructure of the tumours derived from the transformed cells, and confirm their epithelial origin.

### MATERIALS AND METHODS

Explant cultures of adult mouse salivary gland were treated for 24 h with 7,12-dimethylbenzanthracene (DMBA) on Day 3 of culture. Epithelial cell foci developed in the cultures. Five tumour-producing cell lines were established from these foci, 4 after treatment of cells with DMBA, and one which arose without carcinogen treatment. Details of treatment and tissue-culture techniques are given in an earlier paper (Knowles and Franks, 1977). Cells were removed from their containers with 0·25% Pronase (Calbiochem Limited, London) and 1 or $2 \times 10^6$ cells in 0·1 ml of tissue-culture medium were inoculated s.c. into weanling syngeneic hosts. Mice were killed when tumours were about 1 cm in diameter. A total of 48 primary tumours were established from early passage cells of the 5 cell lines. The tumours which developed were retransplanted s.c. using a modified Bashford needle, and portions of each tumour were also minced and suspended in 10% dimethyl sulphoxide in 6% glucose and stored in liquid $N_2$. Tissues from tumours were taken for histology. They were fixed in 5% neutral phosphate-buffered formalin and embedded in paraffin wax. Sections (5–8 $\mu$m) were stained with haematoxylin and eosin (H. & E.); sections from 12 tumours were stained by the following methods to demonstrate mucopolysaccharides: alcian blue, periodic acid–Schiff (ABPAS) (Mowry and Winkler, 1956), phenylhydrazine PAS (Spicer, 1961) and sialidase AB PAS (Gad, 1969). Details and discussion of the above methods are given by Gad (1969).

For electron microscopy, tissue from each tumour was chopped and fixed overnight in 2·5% glutaraldehyde in 0·1M sodium cacodylate buffer at 4°C, rinsed in 0·1M sodium cacodylate buffer at 4°C and post-fixed in Palade's fluid for 1 h over ice. Tissue blocks were dehydrated in graded ethanols, and embedded in Araldite using epoxypropane as transitional solvent. Ultrathin sections were cut on an LKB ultramicrotome, stained with alcoholic uranyl acetate and lead citrate (Reynolds, 1963) and viewed in a Philips 301 microscope.

### RESULTS

Implantation of tissue-culture cells produced 48 primary tumours from the 5 transformed cell lines. There were no significant differences between the tumours from the carcinogen-treated and untreated cell lines. The tumours were firm, with a glistening grey rather gelatinous cut

FIGS. 1–8 are H & E-stained sections of tumours from tissue-culture cells transformed *in vitro*.

FIG. 1.—Varied tumour pattern with large and small acini and solid cords of cells. × 75.
FIG. 2.—Acinar pattern, some dilated tubules and some solid trabeculae in centre. × 225.
FIG. 3.—Anaplastic tumour with a solid cord of cells right of centre. × 225.
FIG. 4.—Area showing squamous metaplasia (right) and anaplastic tumour. × 225.
FIG. 5.—Papillary area. × 225.
FIG. 6.—Cords of darkly staining cells and giant cells (arrowed). × 225.
FIG. 7.—Myxoid area with tumour acini and cords of cells. × 225.
FIG. 8.—Adenocarcinoma (left) and spindle-cell sarcomatous area (right).

Figs. 9–17 are from sections of Araldite-embedded tumours, stained with uranyl acetate and lead citrate.

Fig. 9.—Part of a tumour tubule resembling an intercalated duct. ×2250.

Fig. 10.—Part of the tubule showing a thin cell process of basal cell, separating the luminal cell from the underlying stroma (bottom left). The luminal cell has surface microvilli and junctional complexes (arrowed) near the lumen. ×7500.

Fig. 11.—The base of a tubule cell showing basal lamina and 2 hemidesmosomes. ×56,250.

Fig. 12.—The luminal surface of 2 adjoining tubule cells showing microvilli and associated extracellular electron-dense material, probably surface glycoprotein (arrowed). The lower cell contains membrane-bounded secretory vesicles near the lumen. There is a junctional complex between the cells. ×11,250.

surface. A feature of most of the tumours was the marked differences in morphology in different areas (Figs. 1–3). There were 3 predominant epithelial patterns: 1, tubular (Fig. 2), sometimes with dilated lumina; 2, solid epithelial strands (solid trabecular, Figs. 2 and 3), and 3, anaplastic with irregular masses of ill defined epithelial cells (Figs. 3 and 4). In some tumours there was basal and squamous-cell metaplasia (Fig. 4). Papillary areas were rarely present (Fig. 5). The degree of anaplasia of the cells also varied. In some parts, particularly in the tubular areas, the cells were well differentiated. In some tumours, darkly staining polygonal cells resembling myoepithelial cells and giant cells of similar structure were also found (Fig. 6). The stroma varied in amount. In most tumours the epithelium was closely packed, but in others it was separated by dense hyaline material or a loose myxomatous stroma (Fig. 7) resembling that of myxoid areas found in human "mixed" salivary-gland tumours (Welsh and Meyer, 1968). Almost all the tumours showed central degeneration and a myxoid zone between the necrotic centre and the surviving rim of healthy tissue. One cell line produced tumours with both epithelial and leiosarcomatous components (Fig. 8) the latter being similar in structure to the tumours produced by spontaneously transformed mesenchymal cell lines (Franks, Chesterman and Rowlatt, 1970). Although all tumours had invaded the surrounding muscle and vascular invasion was common, metastases were not found.

Some of the tumour acini contained an acid mucin which stained with alcian blue, and their reaction was abolished by pretreatment with sialidase. The material is therefore an epithelial sialomucin. Acid mucin was also present in the stroma. Some was sialidase-sensitive and probably of epithelial origin. The remainder was sialidase-insensitive, did not react with the phenylhydrazine PAS stain and was probably of stromal origin.

Transplanted tumours, and tumours derived from tissue stored in liquid $N_2$, retained the structure of the primary tumours, except in one carcinoma, in which the second and subsequent transplant generations were much more anaplastic than the primary. The mixed carcinosarcoma produced transplanted tumours similar to the tumour of origin.

*Ultrastructure*

Although each of the 3 predominant epithelial patterns were clearly defined at the light-microscope level, the electron-microscope showed that they shared many features, and that the differences were due to a lack of structural rather than cellular differentiation. The tubular areas (Fig. 9) resembled normal intercalated ducts very closely (Tamarin and Screebny, 1965) and had occasional basal cells (Fig. 10) lying between the surface epithelium and the basal lamina. Although similar in position to myoepithelial cells, these basal cells did not have any of the ultrastructural features of myoepithelial cells. Normal junctional complexes were present and the tubules were surrounded by an almost complete layer of basal lamina, with occasional hemidesmosomes (Fig. 11). The luminal surface had typical epithelial microvilli with central filament bundles, and associated electron-dense extracellular glycoprotein (Fig. 12).

The cells contained the usual cell organelles, but the mitochondria were larger and more irregular than those in normal cells. Most cells had many free ribosomes. Occasionally cells showed evidence of secretion, either with groups of membrane bounded vesicles (Fig. 12) near the luminal surface or with lamellae of endoplasmic reticulum resembling serous acinar cells (Fig. 13). Glycogen deposits were found in some cells.

The solid trabecular (Fig. 14) and anaplastic areas (Fig. 15) differed from the tubular areas in the size of the cell masses, the degree of luminal distention, the absence of secretory activity and a relative loss of cell polarity. Small micro-lumina were present in the solid travecular

FIG. 13.—A tubular cell resembling a serous acinar cell. ×11,250.
FIG. 14.—Solid trabecular area. Small microlumina and surface microvilli can be seen. ×2250.
FIG. 15.—An anaplastic area. ×2250.

Fig. 16.—A squamous area. The cells have bundles of darkly staining filaments (arrowed) and desmosomes (arrowheads). × 11,250.

Fig. 17.—A myxoid area with a central strand of epithelial tumour cells invading stroma (arrowed). There are collagen bundles and fibroblasts on the left. On the right collagen bundles and fibroblasts are separated by loose pale-staining myxoid stroma. × 2250.

cell masses, which were only visible in the electron microscope. In these areas the cells retained junctional complexes and microvilli. Many cell masses were partially surrounded by basal lamina, even in the anaplastic areas. Cells in the squamous areas had many tonofilament bundles and darkly staining desmosomes (Fig. 16). Tumour-cell invasion occurred through the basal lamina, but in some parts the luminal margins of the cells infiltrated the surrounding collagen (Fig. 17).

The myxoid areas (Fig. 17) were made up of masses of amorphous material with scattered bundles of collagen and elastic fibres, surrounding small groups or single cells, some resembling fibroblasts, others probably epithelial, and many which could not be identified with certainty.

### DISCUSSION

In any work on epithelial carcinogenesis *in vitro*, it is important to establish the epithelial nature of the tumours produced, particularly as many mesenchymal tumours from *in vitro*-transformed cells may have an anaplastic epithelial-like pattern (Franks *et al.*, 1970). The structure of the tumours we have described shows beyond question that the cells which had been transformed *in vitro* were epithelial. The sarcomatous element in the mixed carcinosarcoma presumably arose from mesenchymal cells transformed spontaneously or by DMBA treatment.

Although spontaneous salivary-gland tumours occur rarely in mice (Murphy, 1966) these resemble the human basal-cell tumours of the salivary gland (Jao, Keh and Swerdlow, 1976) rather than the more usual "mixed" salivary-gland tumours. The morphology and ultra structure of the tumours derived from the *in vitro* transformed cells are almost identical to the common human tumours, but particularly to their more malignant variants (Welsh and Mayer, 1968; Evans and Cruickshank, 1970). There seems to be little doubt that the human epithelial tumour cells arise from the intercalated ducts (Evans and Cruickshank, 1970) and that the stromal changes are largely due to the release of acinar material, although in some cases neoplastic spindle-cell areas are present which cannot be distinguished from leiomyosarcomas. These resemble the mixed carcinosarcoma which we have described (see for example Figs. 10–54 *et seq.* from Evans and Cruickshank, 1970). Welsh and Meyer (1968) conclude that some human tumours have both mesenchymal and epithelial neoplastic components.

Like the human tumours, the mouse tumours show a wide range of structural differentiation, which would be expected from tumours derived from a multipotential cell. The tumours derived from the cells transformed by DMBA *in vitro* differ in structure from those which arose from *in vivo* treatment (Wigley and Carbonell, 1976). These *in vivo* tumours, which were predominantly squamous, were thought to arise from a more differentiated cell, the granular tubule cell (Wigley and Carbonell, 1976).

### REFERENCES

Evans, R. W. & Cruickshank, A. H. (1970) In *Epithelial Tumours of the Salivary Glands*. Philadelphia: W. B. Saunders. pp. 209, 226.

Franks, L. M., Chesterman, F. C. & Rowlatt, C. (1970) The Structure of Tumours Derived from Mouse Cells after "Spontaneous" Transformation *in vitro*. *Br. J. Cancer*, **24**, 843.

Gad, A. (1969) A Histochemical Study of Human Alimentary Tract Mucosubstances in Health and Disease. I. Normal and Tumours. *Br. J. Cancer*, **23**, 52.

Jao, W., Keh, P. C. & Swerdlow, M. A. (1976) Ultrastructure of the Basal Cell Adenoma of Parotoid Gland. *Cancer, N.Y.*, **37**, 1322.

Knowles, M. & Franks, L. M. (1977) Neoplastic Transformation of Adult Epithelial Cells *in vitro*: Stages in the Progression to Malignancy. *Cancer Res.*, **37**, 3917.

Mowry, R. W. & Winkler, C. H. (1956) The Coloration of Acidic Carbohydrates of Bacteria and Fungi in Tissue Sections with Special Reference to Capsules of *Crytpococcus neoformans*, Pneumococci, and Staphylococci. *Am. J. Path.*, **32**, 628.

Murphy, E. D. (1966) Characteristic Tumors. In *The Biology of the Laboratory Mouse* Ed. Green, E. L. 2nd edn. New York: McGraw-Hill. p. 521.

Reynolds, E. S. (1963) The Use of Lead Citrate at High pH as an Electron-opaque Stain in Electron Microscopy. *J. Cell Biol.*, **17**, 208.

SPICER, S. S. (1961) The Use of Various Cationic Reagents in Histochemical Differentiation of Mucopolysaccharides. *Am. J. clin. Path.*, **36**, 393.

TAMARIN, A. & SCREEBNY, L. M. (1965) The Rat Salivary Gland. A Correlative Study by Light and Electron Microscopy. *J. Morph.*, **117**, 295.

WELSH, R. A. & MEYER, A. T. (1968) Mixed Tumors of Human Salivary Gland. *Archs Path.*, **85**, 433.

WIGLEY, C. B. & CARBONELL, A. W. (1976) The Target Cell in the Chemical Induction of Carcinomas in Mouse Submandibular Gland. *Eur. J. Cancer*, **12**, 737.